# Understanding the Antecedents of the Routine Use of Mobile Health Services: A Person–Technology–Health Framework

**DOI:** 10.3389/fpsyg.2022.879760

**Published:** 2022-06-16

**Authors:** Fanbo Meng, Xitong Guo, Zeyu Peng, Xiaofei Zhang, Kee-hung Lai

**Affiliations:** ^1^School of Business Jiangnan University, Wuxi, China; ^2^Harbin Institute of Technology, Harbin, China; ^3^Business School, East China University of Science and Technology, Shanghai, China; ^4^School of Business, Nankai University, Tianjin, China; ^5^Department of Logistics and Maritime Studies, Hong Kong Polytechnic University, Kowloon, Hong Kong SAR, China

**Keywords:** mHealth services, personal innovativeness in IT, trust, perceived health severity, routine use intention

## Abstract

Although numerous studies have been conducted to understand the antecedents of usage of mobile health (mHealth) services, most of them solely focus on characteristics of mHealth services themselves but neglect taking users’ psychological and health-related factors into consideration. Besides, the comprehensive understanding of what influences users’ routine use intentions regarding mHealth services is lacking. Therefore, this study proposes a person–technology–health framework that underlines how personal factors (e.g., personal innovativeness in IT), technological factors (e.g., trust), and health factors (e.g., perceived health severity) jointly influence individuals’ routine use intentions regarding mHealth services. The proposed research model and related hypotheses were tested based on survey data from 270 respondents. The results indicate that personal innovativeness in IT, trust, and perceived health severity are important for enhancing routine use intention of mHealth services. Specifically, in situations of high perceived health severity, trust relates less positively to routine use intention than personal innovativeness in IT. In contrast, in situations of low perceived health severity, trust relates more positively to routine use intention than personal innovativeness in IT. The research findings extend the existing literature on routine use intention related to mHealth services and provide significant implications for practitioners.

## Introduction

Empowered by mobile information communication technology, mobile health (mHealth) services as emerging health-related information technology can deliver timely and ubiquitous health information and services to individuals based on individual-tailored healthcare needs (Akter and Ray, 2010; Akter et al., 2011). The increasing population with chronic diseases and multi-morbidities in recent years calls for an increase in the routine use of mHealth services. Because mHealth services have shown great potential for improving patient wellbeing, mental health, health management, and distribution of medical resources (Hoque and Sorwar, 2017; Zhao et al., 2017; Oliveira et al., 2021). Furthermore, the application of mHealth services in the diagnosis and treatment of infectious diseases could improve outbreak detection, disease surveillance, and guide a precise response of public health (Wood et al., 2019). However, achieving these outcomes highly depends on users’ sufficient data based on daily interactions (Deng et al., 2014; Zhao et al., 2017). Therefore, to increase both user stickiness and developers’ profits, it is more important to enhance existing users’ day-to-day use as a form of routine use than it is to acquire more mHealth users (Li et al., 2013; Meng et al., 2019b). However, the routine use of mHealth services remains at a lower level, in particular, the daily use rate is 5.7% and the weekly use rate is 30.6% based on a recent work of Knitza et al. (2020). To this end, it is urgent to understand what influences users’ routine use of mHealth services.

Existing literature is abundant in investigating various antecedents of the adoption and uses intention of mHealth services. These include both technological factors (individuals’ evaluations of mHealth), such as perceived usefulness, perceived ease of use, perceived trustworthiness, and perceived value (Sun et al., 2013; Deng et al., 2014; Okazaki et al., 2015; Fox and Connolly, 2018; Alam et al., 2020) and personal factors (individuals’ personality traits) like personal innovativeness in IT (PIIT), technology anxiety, self-efficacy, and privacy concerns (Rai et al., 2013; Deng et al., 2014; Guo et al., 2016; Reychav et al., 2019). In a typical professional setting, such as health services, however, users may exhibit interesting or fundamental differences from ordinary business user groups, in part because of their health conditions (Chau and Hu, 2002; Gorini et al., 2018). The use of mHealth services has also been examined from the perspective of Protection Motivation Theory (PMT), which suggests that users are willing to use mHealth services to improve their health status in order to avoid health threats, such as chronic diseases (Rai et al., 2013; Zhao et al., 2017). These studies and others indicate that health-related factors (individuals’ evaluations of their health conditions), such as perceived physical condition, perceived severity, perceived vulnerability, and health rationality, significantly influence adoption and use intention of mHealth services (Deng et al., 2014; Guo et al., 2015; Zhao et al., 2017; Zhang et al., 2020).

In this light, we consider these three categories of factors to be salient antecedents for predicting individuals’ attitudes and behaviors regarding mHealth services. However, only a limited number of studies in the extant mHealth literature consider how these factors jointly affect the routine use of mHealth services (Deng et al., 2015; Zhao et al., 2017; Meng et al., 2021). Moreover, to the best of our knowledge, no studies integrate and underline the interactions among those factors to comprehensively understand the routine use of mHealth services. It is thus imperative to narrow this research gap by developing a more comprehensive research framework, which can theoretically integrate these factors in order to predict mHealth service adoption and use in general and routine use in particular (Rai et al., 2013; Meng et al., 2019b, 2020).

Drawing upon relevant literature on mHealth services and health informatics, we therefore propose an integrative person–technology–health (PTH) research framework to predict routine use of mHealth services by testing the interaction effects of technological factors, personal factors, and health factors on routine use intention. Accordingly, PIIT, trust, and perceived health severity are theoretically identified as three critical factors of the PTH framework and integrated into a research model. Here, mHealth services refer to healthcare delivery through mobile information technology, which require users’ high level of engagement to access timely personalized health services for improved health conditions (Akter et al., 2013a). Therefore, we believe that the PTH framework is particularly appropriate for predicting routine use of mHealth. Theoretically, our study is one of the first to propose and empirically investigate the combined effects of technological, personal, and health factors on routine use intention of mHealth services based on the PTH framework. In addition, this integrative PTH framework provides insights into other contexts of health-related IT adoption and usage and thus may be valuable in future studies. In practice, mHealth service providers can take advantage of the PTH framework to precisely customize their marketing strategies based on the joint effects of trust, PIIT, and perceived health severity to increase their users’ stickiness as a form of routine use and obtain long-term benefits.

The remainder of this paper proceeds as follows. First, we discuss the previous literature on various factors of the PTH framework. Then, we present the proposed research framework and hypotheses, followed by the research methodology and data analyses. Finally, we report the key findings, implications for research and practice, and limitations of our work.

## Literature Review

### Factors of the PTH Framework

Our study develops the PTH framework based on the mHealth services literature and the health informatics literature. Prior studies of mHealth service adoption and usage were conducted based on a small number of well-worn and conceptually interrelated theories, such as the theory of planned behavior (Ajzen, 1991), technology acceptance model (Davis, 1989; Venkatesh et al., 2002), motivational model (Davis et al., 1992), and the unified theory of acceptance and use of technology (Venkatesh et al., 2003). As shown in Table 1, previous studies mainly examine the effects of technological factors (e.g., perceived usefulness, perceived ease of use, and trust) and personal factors (e.g., perceived behavioral control, computer self-efficacy, and technology anxiety) on mHealth service adoption and usage (Chen et al., 2018; Balapour et al., 2019; Cocosila and Turel, 2019; Alam et al., 2020). On the other hand, studies within the health informatics literature have demonstrated that health factors, such as health status and perceived health conditions, are significantly associated with the use of health IT (Rai et al., 2013; Deng et al., 2014; Cho et al., 2014b; Lagoe and Atkin, 2015; Meng et al., 2021). All these factors can be categorized according to the PTH framework, thus indicating the appropriateness of this conceptual framework for understanding critical antecedents of mHealth service use.

**Table 1:** Factors of the PTH framework in prior studies.

Literature	Topic	Factors of the PTH framework			Dependent variable	Two-way interacti
		Technological factor	Personal factor	Health factor		
Wu et al., 2011	mHealth services	Perceived usefulness; perceived ease of use; perceived service availability	Perceived behavioral control; personal innovativeness in IT;		Behavioral intention	No
Guo et al., 2012	mHealth services	Perceived usefulness; perceived ease of use	Technology anxiety; resistance to change		Adoption intention	No
Cocosila, 2013	mHealth services	Extrinsic motivation; intrinsic motivation; perceived risk	*A priori* attitude toward the activity		Behavioral intention	No
Rai et al., 2013a	mHealth services		Personal innovativeness toward mobile services; socioeconomic status and demographics	Perceived health conditions	Usage intentions and channel preferences	Yes
Akter et al., 2013b	mHealth services	Perceived usefulness; perceived service quality; perceived trust			Continuance intention	No
Cho et al., 2014a	Health application	Perceived usefulness; perceived ease of use	Health consciousness; health information orientation; eHealth literacy; health application use efficacy		Behavioral intention to use	No
Deng et al., 2014	mHealth services	Perceived value	Perceived behavioral control; resistance to change; self-actualization need; technology anxiety	Perceived physical condition	Behavioral intention	No
Shareef et al., 2014	mHealth services	Perceived usefulness; perceived privacy and security; perceived ease of use; perceived compatibility; perceived reliability			mHealth adoption	No
Deng et al., 2015	mHealth services	Information quality; perceived value; trust	Personal health value	Current health status	Use intention	Yes
Guo et al., 2015	mHealth services		Response efficacy; self-efficacy; age; gender	Perceived vulnerability; perceived severity	Behavioral intention	Yes
Okazaki et al., 2015	mHealth monitoring systems	Perceived value; overall quality; net benefits	Prior mobile internet experience		Usage intention	Yes
Guo et al., 2016	mHealth services	Trust	Privacy concern; personalization concern; age		Adoption intention	No
Zhao et al., 2017	mHealth services	Perceived usefulness; perceived ease of use; trust; perceived risk	Perceived behavioral control; age	Perceived vulnerability; perceived severity	Behavioral intention	Yes
Deng et al., 2018	mHealth services	Perceived usefulness; perceived ease of use; trust; perceived risk			Adoption intention	No
Fox and Connolly, 2018	mHealth services	Risk beliefs; trust beliefs	Mhealth self-efficacy; Information seeking experience; health information privacy concerns		Adoption intention	No
Chen et al., 2018	Mobile health application	Perceived usefulness; trust in app	Privacy concern		Continuance intention	Yes
Balapour et al., 2019	mHealth apps	Mobile technology identity	Related IT expertise; self-efficacy		Intention to use	No
Meng et al., 2019b	mHealth services	Argument quality; source credibility	Health consciousness		Routine use intention	Yes
Cocosila and Turel, 2019	mHealth services	Extrinsic motivation; intrinsic motivation; adoption risk; non-adoption risk			Adoption intention	No
Zhang et al., 2020	Mobile monitoring services	Device satisfaction; feedback satisfaction	Emotional attachment	Health rationality	Mobile monitoring services usage	Yes
Meng et al., 2021	mHealth services	Cognitive trust; affective trust	Technology anxiety	Health anxiety	Continuance use intention	Yes

According to our summary of extant literature on mHealth service adoption and usage (see Table 1), three research gaps need to be filled. First, with some exceptions (Deng et al., 2014; Zhao et al., 2017), many studies failed to integrate person, technology, and health factors into a single research framework and simultaneously test their direct effects on mHealth service adoption and use. Second, although some scholars examined either the interaction effects of technological factors and personal factors (Okazaki et al., 2015; Chen et al., 2018) or the interaction effects of personal factors and health factors on mHealth service adoption and use (Rai et al., 2013), most scholars failed to investigate the interactions among all three factors to predict mHealth service adoption and use. Third, to our best knowledge, scant research exists to examine mHealth users’ routine use based on a comprehensive framework involving technological, personal, and health-related factors. In order to fill these research gaps, we intend to integrate all of the aforementioned factors into one single research framework. We test not only the direct effects of personal factors, technological factors, and health factors but also the interaction effects among them on routine use intention, thus further advancing our understanding of users’ routine use intention regarding mHealth services.

### Interaction Between Personal Factors and Technological Factors

The interaction between personal factors and technological factors has been extensively studied in the prior information systems literature. Personal evaluations of technology (e.g., perceived usefulness, perceived ease of use, and trust in IT) are directly derived from the interactions between users and technology. This perspective is supported by the innovation-values fit theory, which refers to the extent to which users perceive that the use of the innovation is congruent with users’ values by assessing “the objective characteristics of an innovation and its socially constructed meaning” (Klein and Sorra, 1996, p. 1063). In other words, an individual’s use of a technology is determined by the level of the fit between the innovation and her/his value (Klein and Sorra, 1996).

The effects of this interaction on mHealth service adoption and usage have been examined by several scholars. Okazaki et al. (2015) examined the moderating effects of users’ experience on perceived value and found that users with prior mobile internet experience will perceive greater value in using an mHealth monitoring system than those without relevant experience, thus leading to a higher probability that these more experienced technology users will use mHealth services. Some scholars selected age and gender as moderators in their mHealth service adoption study, and their results indicated that compared to younger users, middle-aged and older users pay more attention to perceived ease of use and effort expectancy when they are making adoption decisions (Zhao et al., 2017; Nunes et al., 2019). Chen et al. (2018) found that both trust in apps and perceived usefulness are positively associated with continuing use intention regarding mHealth apps, and privacy concerns enhance the effects on continuous use intention. Meng et al. (2019b) found that individuals with higher levels of health consciousness are more willing to rely on source credibility than argument quality to inform their routine use of mHealth services. On the basis of these studies, the extent to which technological factors influence mHealth service adoption and use is contingent on personal factors because mHealth services are defined as personalized and interactive. Therefore, it is necessary to investigate the interaction effects of technological and personal factors to comprehensively understand routine use intention regarding mHealth services.

### Interaction Between Health Factors and Technological Factors

Prior literature indicates that health factors are significantly associated with the use and adoption of health IT. Many of these studies are based on Protection Motivation Theory (PMT), which proposes that individuals’ threat appraisals (e.g., their perceived severity and perceived vulnerability) and coping appraisals (e.g., response efficacy, self-efficacy, and response costs) are two dominant predictors of health behaviors (Rogers, 1975). Perceived severity refers to the seriousness of a specific threat while perceived vulnerability refers to the probability that one will experience harm (Rogers, 1975). Perceived vulnerability and perceived severity are often combined to measure perceived threat; however, the unique relationship of each to health behavior has also been investigated (Witte, 1992; McKinley, 2009; Zhao et al., 2017). Accordingly, individuals with poor health conditions are more likely to use health IT to improve their health status (Houston and Allison, 2002; Baker et al., 2003; Rai et al., 2013; Xiao et al., 2014). Also researching these effects, Guo et al. (2015) found that among women and the elderly, perceived severity and perceived vulnerability strongly influence individuals’ attitudes toward using mHealth services. Zhao et al. (2017) indicated that perceived vulnerability and perceived severity are significant factors in predicting middle-aged and older users’ use of mHealth services.

Researchers have also explored the role of health anxiety: individuals who feel anxious about their health conditions show greater inclination to seek out online health information (Baumgartner and Hartmann, 2011; Lagoe and Atkin, 2015; te Poel et al., 2016). For example, Baumgartner and Hartmann (2011) found that individuals with a high level of health anxiety experience more negative consequences from searching for health information online. However, the findings in this area are inconsistent. For instance, Xue et al. (2012) found that female users’ perceived health conditions have no direct effects on perceived usefulness, perceived ease of use, and perceived compatibility of mHealth apps.

To the best of our knowledge, however, there has been little research on the interaction effects between health factors and technological factors on the use and adoption of mHealth services. There are some exceptions, including Deng et al. (2015), who found that individuals with poor health status (e.g., mental or physical illness) rely more on trusted health information providers and are more eager to use mobile phones to get health information with the purpose of improving their health. Meng et al. (2021) demonstrated that health anxiety increase the effects of cognitive trust but alleviate the effect of affective trust on continuous intention of mHealth services use.

Considering that health services closely relate to people’s life quality (Fichman et al., 2011), individuals with various health conditions may hold various attitudes toward using a target health IT, such as an mHealth service, in the long run. Therefore, it is urgent for mHealth scholars to investigate the interaction effects of health and technological factors on routine use.

### Interaction Between Personal Factors and Health Factors

Studies have indicated that both individuals’ personal and health-related factors are important predictors of health-related behaviors and technology adoption in the health behavior context. Based on the basic tenet of the health belief model (Rosenstock, 1974; Janz and Becker, 1984), the health-promoting behaviors of individuals with different socio-demographic characteristics are determined by their beliefs about health conditions (e.g., perceived severity and perceived susceptibility), perceived benefit, and perceived barriers (Hochbaum et al., 1952). Moreover, personal factors, such as age, gender, race, socioeconomic status, and characteristics, also influence individuals’ health-related behavior (Carroll et al., 2017).

Some scholars examined the interactions of personal and health-related factors in the context of mHealth service adoption and usage. Rai et al. (2013a) indicated that personal innovativeness toward mobile services (PIMS) has positive moderating effects on perceived health conditions comprising perceived healthiness and perceived vulnerability. Moreover, the interactions of consumers’ PIMS and perceived health conditions have significant positive effects on mHealth service usage intentions, assimilation, and channel preferences (Rai et al., 2013). Guo et al. (2015) found that personal factors (e.g., gender and age) have different effects on threat appraisals (perceived vulnerability and perceived severity) and coping appraisals (response efficacy and self-efficacy) in the acceptance of mHealth services. For example, the female and the elderly are more willing to form positive attitudes and accept mHealth services when they perceive higher levels of vulnerability and severity. Similarly, Zhao et al. (2017) found that perceived vulnerability and perceived severity are more significant predictors of mHealth service use among middle-aged and older users. However, the interaction effects of personal and health factors on the routine use of mHealth services still remain unexplored, thus calling for additional research.

## Research Model and Hypotheses

**Figure 1:**
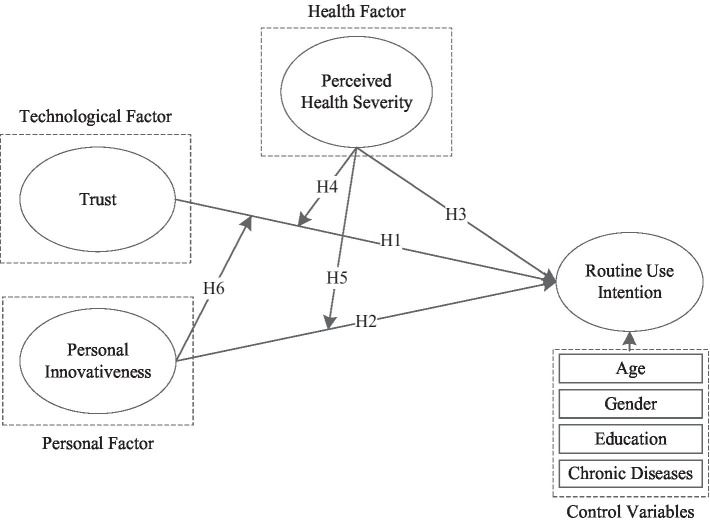
The research model.

According to previous work on the adoption and use of mHealth services, personal factors [e.g., PIIT (Lu, 2014)], technological factors [e.g., trust (Deng et al., 2018)], and health-related factors [e.g., health severity (Or and Karsh, 2009)] have been identified as significant factors influencing mHealth adoption and use. However, interaction effects and combined effects of personal factors, technological factors, and health-related factors on mHealth routine use have not been fully investigated. To comprehensively understand antecedents of mHealth routine use, drawn upon information system and health informatics literature, we propose the PTH research framework (Figure 1) that incorporates constructs of trust, PIIT, and perceived health severity to investigate routine use intention. First, we propose that these three variables have direct effects on routine use intention (H1, H2, and H3). Second, we test the moderating effects of perceived health severity on the associations between two components and routine use intention (H4 and H5). Finally, we test the moderating effect of PIIT on the association between trust and routine use intention (H6).

### Technological Factor: Trust

Trust in technology refers to a person’s judgment or expectation that a given technology’s helpfulness, reliability, and functionality will support them in their work (Thatcher et al., 2011). As trust can reduce risks and uncertainties, it plays a significant role in the adoption of a new IT (Kim and Prabhakar, 2004). Prior studies underscore the fact that trust is a significant prerequisite of social behavior and positively associated with users’ use intentions in multiple contexts: an internet store (Jarvenpaa et al., 1999), purchasing books on the internet (Gefen, 2000), e-government services (Carter and Bélanger, 2005; Lim et al., 2012), e-commerce (Gefen et al., 2003; Pavlou, 2003; Palvia, 2009), and health informatics (McKinley and Ruppel, 2014; Xiao et al., 2014).

Scholars studying mHealth service adoption and usage have found that trust significantly influences users’ intentions to use mHealth services (Deng et al., 2015; Guo et al., 2016; Zhao et al., 2017). For example, Guo et al. (2016) found that trust in mHealth service providers enables the reduction of individuals’ privacy concerns and an increase in adoption intentions. Zhao et al. (2017) and Meng et al. (2019a) indicated that trust is positively associated with the behavioral intention to use mHealth services. In their study, Deng et al. (2018) confirmed trust as an important positive and technical factor predicting users’ adoption intention regarding mHealth services. In the post-adoption stage, perceived trust in mHealth services is proved to have strong effects on satisfaction and continuance intentions (Akter et al., 2013b). Similarly, Meng et al. (2021) found that both cognitive and affective trust strongly affect elderly users’ continuous use intention of mHealth services. Furthermore, considering that mHealth services are credence goods, most people have limited knowledge and experience for forming clear perceptions and beliefs. Based on the above statements, individuals will have greater intention to routinely use a more trusted mHealth service. Thus, we hypothesize that:

*Hypothesis 1*: Trust increases individuals’ routine use intentions regarding mHealth services.

### Personal Factor: PIIT

Personal innovativeness in IT (PIIT) refers to an individual’s willingness to try out any new information technology (Agarwal and Prasad, 1998). PIIT is regarded as the most effective determinant of innovation adoption because it reflects an individual’s natural reaction toward a new technology in multiple adoption domains (Lu, 2014). As personal innovativeness is an individual-specific trait, those who are more innovative are likely to develop positive attitudes toward an innovation and use it (Agarwal and Prasad, 1998). According to Lu et al. (2008), individuals with higher PIIT are more prone to be risk-seeking and may develop more positive intentions to adopt or use an innovation. Among personal psychological factors, PIIT has been widely proved as a critical predictor of behavioral intentions by previous studies on m-commerce (Aldás-Manzano et al., 2009; Zarmpou et al., 2012; Zhao et al., 2012). Prior studies of mHealth services demonstrated that PIIT is positively related to an individual’s intention to use mHealth (Wu et al., 2011; Rai et al., 2013). In addition, innovativeness may play an important role because mHealth services are still in the early stage of development and implementation (Lee, 2016). We therefore believe PIIT is an influencing variable in predicting individuals’ routine use intentions regarding mHealth, and we hypothesize that:

*Hypothesis 2*: PIIT increases individuals’ routine use intentions regarding mHealth services.

### Health Factor: Perceived Health Severity

According to the PMT, perceived vulnerability refers to the probability that one will experience harm, while perceived severity refers to the seriousness of a specific threat (Rogers, 1975). When individuals suffer from health-related threats, they are more likely to use new health IT in order to reduce or avoid those threats (Prentice-Dunn and Rogers, 1986). Prior research also suggests that perceived severity is associated with healthy behavior, such as healthy eating, use of online mental health resources, and use of mobile health services (McKinley, 2009; Sun et al., 2013; McKinley and Ruppel, 2014). Distinct from other services, the eventual purpose of accessing health services through health information technology is to improve individuals’ life quality and health conditions (Or and Karsh, 2009). Therefore, individuals with high health severity or in poor health conditions have stronger willingness to adopt and use health information technology with the aim of alleviating threat of diseases than other health-related factors (DiMatteo et al., 2007). Accordingly, we propose health severity as a primary determinant of adoption and use of mHealth services.

In the context of mHealth services, according to the work of Rai et al. (2013a), people who are afflicted with chronic diseases (e.g., diabetes, heart disease, cancer, high blood pressure, and stroke) are more willing to use mHealth services to manage their health. Guo et al. (2015) demonstrated that users’ perceived vulnerability and perceived severity significantly influence their attitudes toward mHealth services. Zhao et al. (2017) found that elderly users have more concern about their health issues, and they are more likely to use mHealth services to get rid of illness threats and stay healthy. Therefore, we can expect that when the perceived seriousness of a health-related threat is higher, individuals are more prone to routinely use mHealth that can minimize or eliminate the threat. Accordingly, we propose that:

*Hypothesis 3*: Perceived health severity increases individuals’ routine use intentions regarding mHealth services.

### Interaction Effects Among Trust, PIIT, and Perceived Health Severity

Based on our previous discussions, we can expect that an individual with a higher degree of perceived health severity is more willing to try out mHealth and use it routinely, especially when they perceive mHealth can be trusted to improve her or his healthcare outcomes. As a consequence, we propose two-way interaction effects among trust, PIIT, and perceived health severity on routine use intention.

With respect to the interaction effects of trust and perceived health severity on routine use intention, an individual with serious health conditions may choose to use mHealth routinely to reduce the threat of disease and stay healthy. However, these users may not have the level of health literacy that a health professional would have, and health literacy is important in evaluating the information and services provided by mHealth (Anderson and Agarwal, 2011; Cho et al., 2014a). Further, consumers with poor health conditions have a high probability to seek health information through a trustworthy mobile application (Deng et al., 2015), this is because health behavior outcomes are positively determined by health seeking behavior (Anker et al., 2011). In this vein, users have a higher level of health severity may rely on the trust in mHealth services than those with a lower level of health severity. Therefore, to minimize the possibility of uncertainty and risk, these users will be more likely to routinely use a trustworthy mHealth platform. Accordingly, the positive relationship between trust in mHealth and routine use intention can be strengthened by health severity, we propose that:

*Hypothesis 4*: Perceived health severity strengthens the positive association between trust and individuals’ routine use intentions regarding mHealth services.

On the other hand, an individual with a higher level of PIIT is more likely to try out any new health IT (e.g., mHealth) as complement or substitute to in-person health services to avoid or reduce the health-related threat when they perceive higher health severity (Lagoe and Atkin, 2015; Zhao et al., 2017). In this situation, the effect of PIIT on routine use intention regarding mHealth services will be increased by health severity. On this basis, we hypothesize that:

*Hypothesis 5*: Perceived health severity strengthens the positive association between PIIT and individuals’ routine use intentions regarding mHealth services.

With regard to PIIT, users with a higher level of PIIT may easily to evaluate mHealth’s helpfulness, reliability, and functionality while they are using this service. Besides, previous studies have proved that individuals with higher PIIT often have higher levels of technology use (Citrin et al., 2000; Goldsmith, 2001, 2002). Accordingly, an innovative mHealth user can easily develop trust that mHealth services will enhance her/his health outcome and alleviate the disease threat, thus leading to routine use intentions. In such a situation, the positive association between trust in mHealth and routine use intention can be strengthened by PIIT. On this basis, we hypothesize that:

*Hypothesis 6*: PIIT strengthens the positive association between trust and individuals’ routine use intentions regarding mHealth services.

## Methodology

This study is based on a leading mobile health service company, Ciyun. cn, in China. Ciyun health technology company was founded in August 2014. The core product includes “one platform and two applications.” The platform is the data intelligent platform which collects, cleans, converts, and labels personal health data to support two applications for serving medical institutions, enterprises, and the government. The number of users on the Ciyun mHealth service platform was over 2 million by 2020. The functions of the platform include online medical consultation, routine appointments in the out-patient clinic, returning visits, medicine reminders, medical records, real-time positioning, etc. Therefore, this target company was an appropriate site for data collection. The questionnaire is randomly distributed to 292 users through Ciyun mHealth service apps. Permission was obtained, and proper arrangements were made by the management board of Ciyun for the success of data collection.

Following the work of Davis et al. (1989) and Morris and Venkatesh (2000), we provided participants an introduction regarding mHealth services before they completing the questionnaire. Participants completed a survey that included the central variables in this study as well as demographics and control measures. We adapted commonly used measures from previous studies with the aim of promoting content validity. The measures of trust were adapted from the work of Gefen et al. (2003), and the measures for PIIT were adapted from work by Agarwal and Prasad (1998). The perceived health severity scale was adapted from the work of Johnston and Warkentin (2010). The measures for routine use intentions were adapted from work by Sundaram et al. (2007). Each item was measured on a 7-point Likert scale. The constructs and measurements of constructs are presented in Multimedia Appendix A.

After we developed the preliminary questionnaire, we sent it to two mHealth scholars for revision, and we also revised some questions based on the feedback from a pretest with 20 doctoral students. Finally, of 292 questionnaires, and 270 valid ones were obtained for a response rate of 92.5%. Among these participants, approximately 46% were males and 54% females. Approximately 70% of the participants were aged 20–40. About 39% of them had attended university and above. About 16.7% of the participants had chronic diseases. The demographic profile of the respondents is summarized in Table 2.

**Table 2:** Demographic profile of the respondents.

Characteristics	Statistic	
	*N*	Percentage
Gender		
Male	125	46.29
Female	145	53.70
Age		
20–30 years	120	44.44
31–40 years	69	25.56
41–50 years	38	14.07
51–60 years	43	15.93
Educational Level		
Primary school	2	0.74
Secondary school	75	27.77
Pre-university	88	32.59
University	71	26.29
Postgraduate	34	12.59
Chronic disease		
Yes	45	16.66
No	225	83.33

## Analysis and Results

The proposed research framework is tested by the partial least squares structural equation modeling (PLS-SEM) because this technique has several advantages. First, PLS-SEM is more appropriate than covariance-based structural equation modeling (CB-SEM) for analyzing a much more complex model and complicated interaction items (Shiau and Chau, 2016; Hair et al., 2019; Khan et al., 2019; Shiau et al., 2019). Second, compared to CB-SEM, PLS-SEM is more suitable for our study comprising more formative constructs and aiming to conduct exploratory research for theory development (Gefen et al., 2011; Shiau and Chau, 2016; Hair et al., 2019; Khan et al., 2019; Shiau et al., 2019). Accordingly, PLS-SEM is adopted for analyzing our research model. The measurement model was first examined to check its appropriateness. Subsequently, the structural model was analyzed to test the proposed hypotheses (Hair et al., 1998). The reliability, convergent validity, and discriminant validity were examined as indicators of the appropriateness of the measurement model (Fornell and Larcker, 1981). Following the work of Fornell and Larcker (1981), reliability was assessed by examining Cronbach’s alpha, composite reliability (CR), and average variance extracted (AVE). Furthermore, it is uncertain whether the combined effects of all these factors play a role in explaining routine use intentions. To address this, we conducted a post-hoc analysis by proposing a three-way interaction to examine the combined effects of trust, PIIT, and perceived health severity on routine use intentions. To examine the three-way interaction effect on intentions to routinely use mHealth, we conducted a t-test that validated its value.

Considering all self-reported measurement scales may lead to a common method bias that could threaten the validity of our results, we conducted an assessment of common method bias (Podsakoff et al., 2003). Following the work of Harman (1976), we did this using Harman’s single factor test. The results showed that all factors explained 74.5% of the variance and the first factor accounted for 37.2% of the variance. Informed by the work of McFarlin and Sweeney (1992), we believe that common method bias is unlikely to be a serious concern in this study.

### The Measurement Model

In the measurement model, the reliability, convergent validity, and discriminant validity were examined as indicators of the appropriateness of the measurement model (Fornell and Larcker, 1981). Following the work of Fornell and Larcker (1981), reliability was assessed by examining Cronbach’s alpha, composite reliability (CR) and average variance extracted (AVE). The value of Cronbach’s alpha was higher than the suggested value of 0.70, thus indicating sufficient reliability. The values of CR (0.852 to 0.920) and AVE (0.658 to 0.793) were above the threshold values of 0.70 and 0.50, respectively, (Chin, 1998). Thus, all indicators indicated acceptable construct reliability (Fornell and Larcker, 1981). The convergent validity was examined by means of assessing whether all the item loadings of each construct were above the threshold value of 0.70 suggested by Chin (1998). The results in Table 3 indicated that the values of the loadings of all items were higher than 0.70, thereby indicating good convergent validity. The discriminant validity was found to be acceptable because the results indicated the loadings of all items were above their cross-loadings on other constructs, and the correlations of any two constructs were smaller than the square roots of the AVE of each construct. The correlations and discriminant validity of all constructs are presented in Tables 3, 4.

**Table 3:** Correlations and discriminant validity.

Construct	Mean	Standard deviation	Cronbach’s alpha	Composite reliability	AVE	RUI	TRU	PIIT	PHS
RUI	5.06	1.108	0.863	0.916	0.785	**0.886**			
TRU	5.38	1.050	0.876	0.915	0.729	0.578	**0.854**		
PIIT	5.61	1.192	0.869	0.920	0.793	0.318	0.354	**0.890**	
PHS	3.80	1.830	0.745	0.852	0.658	0.197	0.081	0.018	**0.811**

AVE = Average variance extracted; RUI = Routine use intention; TRU = Trust; PIIT = Personal innovativeness in IT; PHS = Perceived health severity.

**Table 4:** Loadings and cross-loadings for measurement items.

	Routine use intention (RUI)	Trust	Personal innovativeness (PIIT)	Perceived health severity (PHS)
RUI1	**0.880**	0.531	0.305	0.172
RUI2	**0.901**	0.514	0.282	0.187
RUI3	**0.876**	0.491	0.256	0.165
Trust1	0.489	**0.840**	0.311	−0.003
Trust2	0.463	**0.849**	0.333	0.058
Trust3	0.534	**0.878**	0.289	0.049
Trust4	0.484	0.848	0.281	0.174
PIIT1	0.280	0.334	**0.890**	0.030
PIIT2	0.277	0.333	**0.893**	0.015
PIIT3	0.291	0.281	**0.889**	0.005
PHS1	0.149	0.093	0.035	**0.828**
PHS2	0.133	0.050	−0.049	**0.824**
PHS3	0.186	0.054	0.045	**0.781**

RUI = Routine use intention; TRU = Trust; PIIT = Personal innovativeness in IT; PHS = Perceived health severity.

### The Structural Model

**Figure 2:**
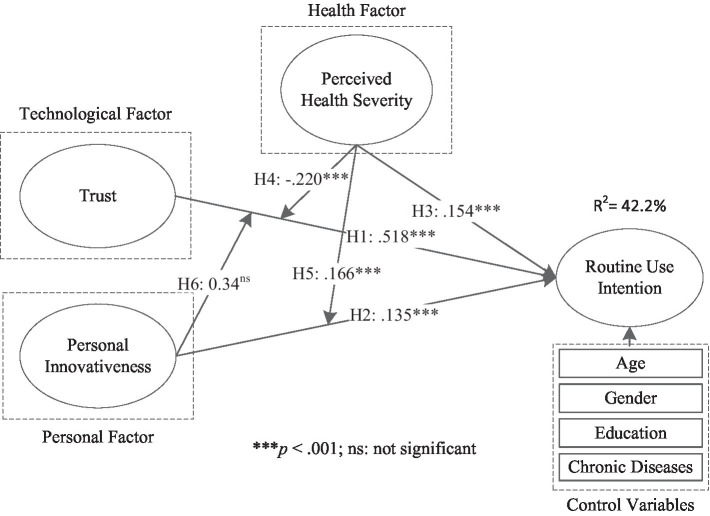
PLS results.

The structural model was assessed by checking the significance of path coefficients (β) between various factors. The PLS results of all proposed relationships are reported in Figure 2. To better examine the interaction effects of these three variables on routine use intentions, we conducted a two-stage criterion in the model analysis. First, the direct effects of trust, PIIT, and perceived health severity on routine use intention were tested in Model 1. In Model 2, the interaction effects among these three variables on routine use intention were tested based on Model 1.

The PLS results for these two models are shown in Table 5. In Model 1, trust (*β* = 0.518, *t* = 10.586, *P*<0.001), PIIT (*β* = 0.135, *t* = 2.383, *p* = 0.008), and perceived health severity (*β* = 0.154, *t* = 3.971, *P*<0.001) were found to have significant effects on routine use intentions. Thereby, H1, H2, and H3 were supported. These three variables combined can explain 37.5% of the variance in routine use intentions. In Model 2, contrary to our hypothesis, perceived health severity was found to have a negative moderating effect on the association between trust and routine use intentions (*β* = −0.220, *t* = 4.176, *P*<0.001), and hence H4 was not supported. Perceived health severity was found to have a positive moderating effect on the association between PIIT and routine use intentions (*β* = 0.166, *t* = 3.498, *P*<0.001), thus supporting H5. However, PIIT had no significant moderating effect on the association between trust and routine use intention (*β* = 0.034, *t* = 0.578, *p* = 0.280). Therefore, H6 was not supported. With respect to the results of control variables, gender (*β* = −0.045, *t* = 0.566, *p* = 0.285), education (*β* = −0.047, *t* = 0.549, *p* = 0.481), chronic diseases (*β* = −0.107, *t* = 1.471, *p* = 0.457), and age (*β* = 0.057, *t* = 0.753, *p* = 0.477) had no significant effects on routine use intention regarding mHealth services. Compared with Model 1, Model 2 (42.2%) explains 4.7% more variance in routine use intentions. The results of each hypothesis are summarized in Table 6.

**Table 5:** The results of the structural equation model (SEM).

Path	Model 1	Model 2
TRU → RUI	0.518^***^	0.484^***^
PIIT→RUI	0.135^**^	0.172^***^
PHS → RUI	0.154^***^	0.140^***^
TRU^*^PHS → RUI		−0.220^***^
PIIT^*^PHS → RUI		0.166^***^
TRU^*^PIIT→RUI		0.034^ns^
*R* ^2^	0.375	0.422
*R*^2^ Change		0.047

RUI, Routine use intention, TRU, Trust, PIIT, Personal innovativeness in IT, PHS, Perceived health severity.

*** *p* < 0.001;

** *p* < 0.01; and

ns, not significant.

**Table 6:** Summary of Results.

Hypothesis description	Result
H1: Trust increases individuals’ routine use intentions regarding mHealth services	Supported
H2: PIIT increases individuals’ routine use intentions regarding mHealth services	Supported
H3: Perceived health severity increases individuals’ routine use intentions regarding mHealth services	Supported
H4: Perceived health severity has a positive moderating impact on the association between trust and individuals’ routine use intentions regarding mHealth services	Not supported
H5: Perceived health severity has a positive moderating impact on the association between PIIT and individuals’ routine use intentions regarding mHealth services	Supported
H6: PIIT has a positive moderating impact on the association between trust and individuals’ routine use intentions regarding mHealth services	Not supported

### *Post-hoc* Analysis

Although trust, PIIT, and perceived health severity successfully explain a significant portion of the variance in routine use intentions regarding mHealth services (42.2%), the three-way interaction effects of these three factors on routine use intention remain underexplored. Therefore, we conducted a post-hoc analysis to examine the combined effects of trust, PIIT, and perceived health severity on routine use intentions.

We hypothesized that perceived health severity would influence the interaction effects of trust and PIIT on individuals’ intention to routinely use mHealth. In situations of low perceived health severity and in individuals with low PIIT, trust may relate more positively to routine use intentions. This is because when the perceived seriousness of a health-related threat is low, individuals with low PIIT may rely on trust to determine their routine use intentions. Such individuals may spend time evaluating the performance of mHealth services, and they may be more likely to make rational behavioral decisions regarding mHealth. In contrast, when individuals with high PIIT perceive their health severity as high, the association between trust and routine use intention would be weaker. This is because such individuals may routinely use mHealth services regardless of the trustworthiness of those services, as they may be more willing to engage in security behavior to reduce the seriousness of the health-related threat. Therefore, they are more likely to make irrational behavioral decisions regarding mHealth services. This supposition is supported by previous studies (Prentice-Dunn and Rogers, 1986; Rai et al., 2013; Zhao et al., 2017). The three-way interaction of trust, PIIT, and perceived health severity had a statistically significant positive effect on implementation (*β* = 0.139, *t* = 3.047, *p* < 0.01) and explained 44.2% of the variance in intentions to routinely use mHealth.

## Discussion and Implications

### Key Findings

There are three key findings from this study. First, consistent with previous studies on mHealth services (Rai et al., 2013; Zhao et al., 2017), we found that both trust and PIIT positively influence routine use intentions. Furthermore, we found that trust has primary explanatory power over PIIT. This affirms the value of trust theory, in which the health information asymmetry between health professionals and normal users helps to explain the adoption and use of mHealth services. Additionally, perceived health severity has a positive impact on routine use intentions. This shows that when users believe that they are more likely to suffer harm from a serious disease, they will tend to use mHealth services routinely to avoid or reduce the threat. This finding is also supported by prior studies (Sun et al., 2013; Guo et al., 2015).

**Figure 3:**
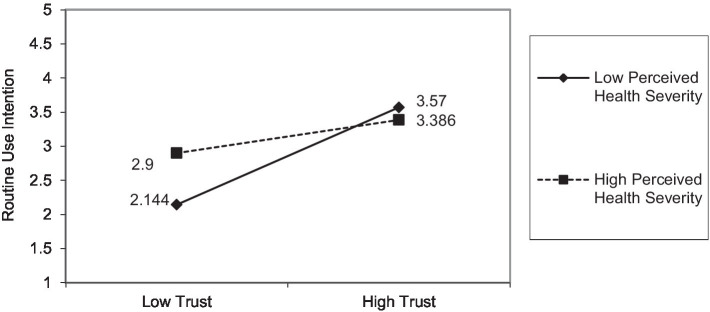
Effects of perceived health severity on the relationship between trust and routine use intention.

**Figure 4:**
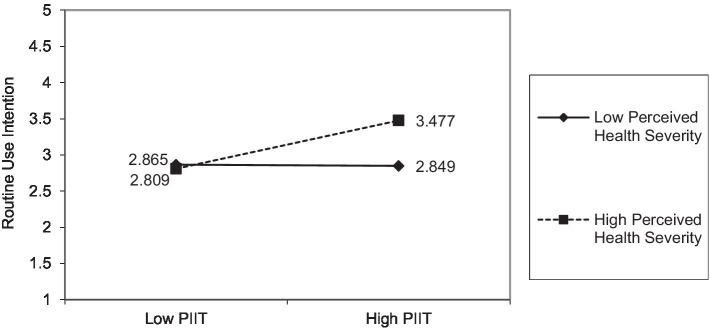
Effects of perceived health severity on the relationship between PIIT and routine use intention.

Second, perceived health severity weakens the effects of trust but strengthens the effects of PIIT on routine use intention. Figure 3, which shows the effects of trust, reflects a large difference in routine use intention under low perceived health severity ( ${\mathrm{RUI}}_{\mathrm{low}}^{\mathrm{trust}}$ = 2.144 vs. $RUI_{high}^{trust}$ = 3.570) and a relatively small difference under high perceived health severity ( ${\mathrm{RUI}}_{\mathrm{low}}^{\mathrm{trust}}$ = 2.900 vs. $RUI_{high}^{trust}$ = 3.386). However, Figure 4, which shows the effects of PIIT, indicates a large difference in routine use intention under high perceived health severity ( ${\mathrm{RUI}}_{\mathrm{low}}^{\mathrm{PIIT}}$ = 2.809 vs. ${\mathrm{RUI}}_{\mathrm{high}}^{\mathrm{PIIT}}$ = 3.477) and a relatively small difference under low perceived health severity ( ${\mathrm{RUI}}_{\mathrm{low}}^{\mathrm{PIIT}}$ = 2.865 vs. ${\mathrm{RUI}}_{\mathrm{high}}^{\mathrm{PIIT}}$ = 2.849). These results indicate that when perceived health severity is at a higher level, PIIT plays a more significant role than trust in enhancing routine use intention. Moreover, the interaction effect between trust and perceived health severity on routine use intention is significant but negative, which is inconsistent with hypothesis 4. This controversial result could be explained by the basic tenet of protection motivation theory (Rogers, 1975). In other words, a higher level of perceived health severity will make individuals feel more anxious about their health conditions and unsafe in the face of the significant threat. Therefore, individuals with higher health severity will try out any new health-related technology (e.g., mHealth services), regardless of its trustworthiness, that can prevent or reduce the threat.

Third, we found that a three-way interaction of trust, PIIT, and perceived health severity affected routine use intention of mHealth services. This indicates that when individuals perceive health severity as low, trust plays a more important role than PIIT in predicting routine use intention. Under the condition of low severity, users tend to make rational health-related decisions. In contrast, when individuals perceive their health severity as high, those with high PIIT will actively engage in routine use of mHealth services even when this innovation is not trustworthy. In this situation, users tend to make irrational health-related decisions.

### Theoretical and Practical Implications

This study can contribute to the mHealth literature in several ways. First, we are one of the first to propose a person–technology–health (PTH) research framework to facilitate a comprehensive understanding of the routine use of mHealth services. Consistent with previous studies (Deng et al., 2015; Zhao et al., 2017; Zhang et al., 2020; Meng et al., 2021), the findings confirm the significant roles of technological factors, personal factors, and health factors in predicting mHealth service adoption and use in general and routine use in particular. More importantly, although prior studies have examined the two-way interaction effect of personal, technological, and health factors on mHealth adoption and use (Deng et al., 2015; Chen et al., 2018; Meng et al., 2019b; Alam et al., 2020), this study, for the first time, tested the three-way interaction effect of aforementioned factors. The findings shed light on the role of personal factors and health factors in influencing the effects of technological factors to various degrees. Therefore, this PTH research framework can address interaction effects in a way that complements traditional adoption theory. Our PTH framework can be adapted by future researchers investigating the adoption and use of specific health IT, such as mHealth services.

Second, this study highlights the difference between mHealth services and other IT by investigating the role of health factors. In contrast to most previous studies focusing solely on the effects of technological factors and personal factors (Hoque and Sorwar, 2017; Chen et al., 2018), this study extends prior research by introducing the role of health factors and exploring the combined effects of technological factors, personal factors, and health factors. Considering the fact that using mHealth services is seen as not only an ordinary IT use behavior but also a health-relevant behavior, this study can provide novel insight into mHealth adoption and usage through illuminating the moderating effect of health factors (e.g., perceived health severity) on technological factors and personal factors. For example, users’ rational or irrational decision-making processes regarding routine mHealth use are to some extent determined by perceived health severity. By taking advantage of such health factors, future studies could shed yield more interesting findings.

Several practical implications can also be derived from the study. First, trust, PIIT, and perceived health severity are found to be significant in promoting users’ routine use intentions of mHealth services. This implies that mHealth service providers should not only develop relevant strategies to improve their services’ technological factors (e.g., trustworthiness, perceived usefulness, and perceived ease of use), but they also need to pay more attention to their targeting users’ personal factors and health factors. Such attention would allow developers to comprehensively understand the antecedents of routine use intentions and focus service development in a way that increases the likelihood of routine use.

Second, our study indicates that perceived health severity has a negative impact on the positive relationship between trust and routine use intention but strengthens the positive relationship between PIIT and routine use intention. Although technological factors (e.g., trust) and personal factors (e.g., PIIT) are critical predictors of routine use intention related to mHealth services, their impacts will be moderated to various degrees in the presence of health factors. With this understanding, providers may be motivated to acquire users’ personal health-related data (e.g., health records, disease history, family heredity history, and disease types). Such data acquisition would aim to differentiate consumers who may face the same health-relevant threat but at different levels, which would allow developers to adopt more user-centric strategies based on users’ health factors, thus increasing users’ routine use and sustaining the company’s development. Overall, the findings of this study could benefit providers of mHealth services by providing the PTH framework, which would allow providers to comprehensively understand how users’ personal traits influence the way they evaluate mHealth services when they are threatened with a health condition.

### Limitations

Our research has several limitations. First, as our representatives of technological, personal, and health factors, we only choose trust, PIIT, and perceived health severity. Other factors may generate more interesting results and increase the explanatory power of the PTH framework. Second, since we collected data in China, the generalizability of this study to other cultural contexts is limited. In western countries, for instance, mHealth users may exhibit different levels of PIIT and perceived health severity. Future studies should validate the research model in other cultural contexts to ensure the validity of the findings. Third, while our study uses a cross-sectional design, which is limited in its ability to draw causal inferences, future researchers could erase this limitation by using a longitudinal design.

## Conclusion

This study proposes the PTH framework as a way to understand users’ routine use of mHealth services by exploring how technological factors (e.g., trust), personal factors (e.g., PIIT), and health factors (e.g., perceived health severity) combine to influence routine use intention. This synthesized PTH research framework provides a greater understanding of the complex and dynamic interactions that influence routine use intentions of mHealth users. Our results show that routine use intentions are significantly associated with trust, PIIT, and perceived health severity, as three components of the PTH framework. Specifically, we found that trust has much more explanatory power than PIIT and perceived health severity. In situations of low perceived health severity, trust will increase the routine use intention of users with low PIIT rather than those with high PIIT. In situations of high perceived health severity, trust plays a less important role in predicting routine use intention for individuals with high rather than low PIIT. The extant literature on mHealth services has provided limited knowledge that is related to the combined effects of technological factors, personal factors, and health factors. Thus, our study not only extends the existing mHealth literature but also provides significant practical implications for providers of mHealth services.

## Data Availability Statement

The raw data supporting the conclusions of this article will be made available by the authors, without undue reservation. Queries and requests to access materials should be directed to the corresponding author.

## Ethics Statement

The studies involving human participants were reviewed and approved by Harbin Institute of Technology School of Management. The patients/participants provided their written informed consent to participate in this study.

## Author Contributions

FM: conceptualization, methodology, and writing—original draft. XG: conceptualization, and writing—review and editing. ZP: methodology and writing—original draft. XZ: writing—review and editing. K-hL: writing—review and editing, and supervision. All authors contributed to the article and approved the submitted version.

## Funding

This study was partially funded by the National Natural Science of China (72001094, 72071054, 71531007, 71871074, and 71871073).

## Conflict of Interest

The authors declare that the research was conducted in the absence of any commercial or financial relationships that could be construed as a potential conflict of interest.

## Publisher’s Note

All claims expressed in this article are solely those of the authors and do not necessarily represent those of their affiliated organizations, or those of the publisher, the editors and the reviewers. Any product that may be evaluated in this article, or claim that may be made by its manufacturer, is not guaranteed or endorsed by the publisher.

## Appendix A

Research Constructs and Items

**Routine Use Intention:** (Sundaram et al., 2007)
RUI 1. I predict I will incorporate mHealth services into my regular life schedule.
RUI 2. mHealth services will be pretty much integrated as part of my normal life routine.
RUI 3. mHealth services will be a normal part of my life.
**Trust:** (Gefen et al., 2003)
PU1. I know that mHealth services are honest.
PU2. I know that mHealth services care about their customers.
PU3. I know that mHealth services are not opportunistic.
PU4. I know that mHealth services are predictable.
**Personal Innovativeness in Information Technology:** (Agarwal and Prasad, 1998)
PIIT1. I am willing to try new information technologies.
PIIT2. I think it is very interesting to try new information technologies.
PIIT3. I enjoy trying new information technologies.
**Perceived Health Severity:** (Johnston and Warkentin, 2010)
PHS1. If I were affected by a disease, it would be severe.
PHS2. If I were affected by a disease, it would be serious.
PHS3. If I were affected by a disease, it would be significant.

## References

[ref1] Agarwal R. Prasad J. (1998). A conceptual and operational definition of personal innovativeness in the domain of information technology. Inf. Syst. Res. 9, 204–215.

[ref2] Ajzen I. (1991). The theory of planned behavior. Organ. Behav. Hum. Decis. Process. 50, 179–211. doi: 10.1016/0749-5978(91)90020-T

[ref3] Akter S. D’Ambra J. Ray P. (2013a). Development and validation of an instrument to measure user perceived service quality of Mhealth. Inf. Manag. 50, 181–195. doi: 10.1016/j.im.2013.03.001

[ref4] Akter S. D’Ambra J. Ray P. (2011). Trustworthiness in Mhealth information services: An assessment of a hierarchical model with mediating and moderating effects using partial least squares (Pls). J. Am. Soc. Inf. Sci. Technol. 62, 100–116. doi: 10.1002/asi.21442

[ref5] Akter S. Ray P. (2010). Mhealth-an ultimate platform to serve the Unserved. Yearb. Med. Inform. 2010, 94–100. doi: 10.1055/s-0038-1638697 20938579

[ref6] Akter S. Ray P. D’Ambra J. (2013b). Continuance of Mhealth Services at the Bottom of the pyramid: The roles of service quality and trust. Electron. Mark. 23, 29–47. doi: 10.1007/s12525-012-0091-5

[ref7] Alam M. Z. Hoque M. R. Hu W. Barua Z. (2020). Factors influencing the adoption of Mhealth Services in a Developing Country: a patient-centric study. Int. J. Inf. Manag. 50, 128–143. doi: 10.1016/j.ijinfomgt.2019.04.016

[ref8] Aldás-Manzano J. Ruiz-Mafé C. Sanz-Blas S. (2009). Exploring individual personality factors as drivers of M-shopping acceptance. Ind. Manag. Data Syst. 109, 739–757. doi: 10.1108/02635570910968018

[ref9] Anderson C. L. Agarwal R. (2011). The digitization of healthcare: boundary risks, emotion, and consumer willingness to disclose personal health information. Inf. Syst. Res. 22, 469–490. doi: 10.1287/isre.1100.0335

[ref10] Anker A. E. Reinhart A. M. Feeley T. H. (2011). Health information seeking: a review of measures and methods. Patient Educ. Couns. 82, 346–354. doi: 10.1016/j.pec.2010.12.008 21239134

[ref11] Baker L. Wagner T. H. Singer S. Bundorf M. K. (2003). Use of the internet and E-mail for health care information: results from a National Survey. JAMA 289, 2400–2406. doi: 10.1001/jama.289.18.2400 12746364

[ref12] Balapour A. Reychav I. Sabherwal R. Azuri J. (2019). Mobile technology identity and self-efficacy: implications for the adoption of clinically supported Mobile health apps. Int. J. Inf. Manag. 49, 58–68. doi: 10.1016/j.ijinfomgt.2019.03.005

[ref13] Baumgartner S. E. Hartmann T. (2011). The role of health anxiety in online health information search. Cyberpsychol. Behav. Soc. Netw. 14, 613–618. doi: 10.1089/cyber.2010.0425, PMID: 21548797

[ref14] Carroll J. K. Moorhead A. Bond R. LeBlanc W. G. Petrella R. J. Fiscella K. (2017). Who uses Mobile phone health apps and does use matter? a secondary data analytics approach. J. Med. Internet Res. 19:e125. doi: 10.2196/jmir.5604 28428170 PMC5415654

[ref15] Carter L. Bélanger F. (2005). The utilization of E-government services: citizen trust, innovation and acceptance factors. Inf. Syst. J. 15, 5–25. doi: 10.1111/j.1365-2575.2005.00183.x

[ref16] Chau P. Y. Hu P. J.-H. (2002). Investigating healthcare professionals’ decisions to accept telemedicine technology: an empirical test of competing theories. Inf. Manag. 39, 297–311. doi: 10.1016/S0378-7206(01)00098-2

[ref17] Chen Y. Yang L. Zhang M. Yang J. (2018). Central or peripheral? Cognition elaboration cues’ effect on users’ continuance intention of Mobile health applications in the developing markets. Int. J. Med. Inform. 116, 33–45. doi: 10.1016/j.ijmedinf.2018.04.008 29887233

[ref18] Chin W. W. (1998). The partial least squares approach to structural equation modeling. Mod. Methods Bus. Res. 295, 295–336.

[ref19] Cho J. Park D. Lee H. E. (2014a). Cognitive factors of using health apps: systematic analysis of relationships among health consciousness, health information orientation, Ehealth literacy, and health app use efficacy. J. Med. Internet Res. 16:e125. doi: 10.2196/jmir.3283, PMID: 24824062 PMC4035139

[ref20] Cho J. Quinlan M. M. Park D. Noh G.-Y. (2014b). Determinants of adoption of smartphone health apps among college students. Am. J. Health Behav. 38, 860–870. doi: 10.5993/AJHB.38.6.8, PMID: 25207512

[ref21] Citrin A. V. Sprott D. E. Silverman S. N. Stem D. E. (2000). Adoption of internet shopping: the role of consumer innovativeness. Ind. Manag. Data Syst. 100, 294–300. doi: 10.1108/02635570010304806

[ref22] Cocosila M. (2013). Role of user a priori attitude in the acceptance of Mobile health: An empirical investigation. Electron. Mark. 23, 15–27. doi: 10.1007/s12525-012-0111-5

[ref23] Cocosila M. Turel O. (2019). Adoption and non-adoption motivational risk beliefs in the use of Mobile Services for Health Promotion. Internet Res. 29, 846–869. doi: 10.1108/IntR-04-2018-0174

[ref24] Davis F. D. (1989). Perceived usefulness, perceived ease of use, and user acceptance of information technology. MIS Q. 13, 319–340. doi: 10.2307/249008

[ref25] Davis F. D. Bagozzi R. P. Warshaw P. R. (1989). User acceptance of computer technology: A comparison of two theoretical models. Manag. Sci. 35, 982–1003. doi: 10.1287/mnsc.35.8.982

[ref26] Davis F. D. Bagozzi R. P. Warshaw P. R. (1992). Extrinsic and intrinsic motivation to use computers in the Workplace1. J. Appl. Soc. Psychol. 22, 1111–1132. doi: 10.1111/j.1559-1816.1992.tb00945.x

[ref27] Deng Z. Hong Z. Ren C. Zhang W. Xiang F. (2018). What predicts patients’ adoption intention toward Mhealth Services in China: empirical study. JMIR 6:316. doi: 10.2196/mhealth.9316 PMC6135967 30158101

[ref28] Deng Z. Liu S. Hinz O. (2015). The health information seeking and usage behavior intention of Chinese consumers through Mobile phones. Inf. Technol. People 28, 405–423. doi: 10.1108/ITP-03-2014-0053

[ref29] Deng Z. Mo X. Liu S. (2014). Comparison of the middle-aged and older users’ adoption of Mobile health Services in China. Int. J. Med. Inform. 83, 210–224. doi: 10.1016/j.ijmedinf.2013.12.002, PMID: 24388129

[ref30] DiMatteo M. R. Haskard K. B. Williams S. L. (2007). “Health Beliefs, Disease Severity, and Patient Adherence: a Meta-Analysis,” United States: Lippincott Williams & Wilkinspp. 521–528. 10.1097/MLR.0b013e318032937e 17515779

[ref31] Fichman R. G. Kohli R. Krishnan R. (2011). Editorial overview-the role of information Systems in Healthcare: current research and future trends. Inf. Syst. Res. 22, 419–428. doi: 10.1287/isre.1110.0382

[ref32] Fornell C. Larcker D. F. (1981). Evaluating structural equation models with unobservable variables and measurement error. J. Mark. Res. 18, 39–50. doi: 10.1177/002224378101800104

[ref33] Fox G. Connolly R. (2018). Mobile health technology adoption across generations: narrowing the digital divide. Inf. Syst. J. 28, 995–1019. doi: 10.1111/isj.12179

[ref34] Gefen D. (2000). E-commerce: The role of familiarity and trust. Omega 28, 725–737. doi: 10.1016/S0305-0483(00)00021-9

[ref35] Gefen D. Karahanna E. Straub D. W. (2003). Trust and tam in online shopping: an integrated model. MIS Q. 27, 51–90. doi: 10.2307/30036519

[ref36] Gefen D. Straub D. W. Rigdon E. E. (2011). An update and extension to Sem guidelines for Admnistrative and social science research. MIS Q. 35, 3–14. doi: 10.2307/23044042

[ref37] Goldsmith R. E. (2001). Using the Domain Specific Innovativeness Scale to Identify Innovative Internet Consumers. Internet Res. 11, 149–158. doi: 10.1108/10662240110695098

[ref38] Goldsmith R. E. (2002). Explaining and predicting consumer intention to purchase over the internet: an exploratory study. J. Mark. Theory Pract. 10, 22–28. doi: 10.1080/10696679.2002.11501913

[ref39] Gorini A. Mazzocco K. Triberti S. Sebri V. Savioni L. Pravettoni G. (2018). A P5 Approach to M-Health: Design Suggestions for Advanced Mobile Health Technology. Front. Psychol. 9:2066. doi: 10.3389/fpsyg.2018.02066 30429810 PMC6220651

[ref40] Guo X. Han X. Zhang X. Dang Y. Chen C. (2015). Investigating M-health acceptance from a protection motivation theory perspective: gender and age differences. Telemed. e-Health 21, 661–669. doi: 10.1089/tmj.2014.0166, PMID: 25919800

[ref41] Guo X. Sun Y. Wang N. Peng Z. Yan Z. (2012). The dark side of elderly acceptance of preventive Mobile health Services in China. Electron. Mark. 23, 49–61. doi: 10.1007/s12525-012-0112-4

[ref42] Guo X. Zhang X. Sun Y. (2016). The privacy–personalization paradox in mHealth services acceptance of different age groups. Electron. Commer. Res. Appl. 16, 55–65. doi: 10.1016/j.elerap.2015.11.001

[ref43] Hair J. F. Anderson R. E. Tatham R. L. William C. (1998). “Multivariate Data Analysis.” Upper Saddle River, NJ: Prentice Hall.

[ref44] Hair J. F. Risher J. J. Sarstedt M. Ringle C. M. (2019). When to use and how to report the results of Pls-Sem. Eur. Bus. Rev. 31, 2–24. doi: 10.1108/EBR-11-2018-0203

[ref45] Harman H. H. (1976). Modern Factor Analysis: London: University of Chicago press.

[ref46] Hochbaum G. Rosenstock I. Kegels S. (1952). “Health Belief Model,” United States: United States Public Health Service.

[ref47] Hoque R. Sorwar G. (2017). Understanding factors influencing the adoption of Mhealth by the elderly: An extension of the Utaut model. Int. J. Med. Inform. 101, 75–84. doi: 10.1016/j.ijmedinf.2017.02.002, PMID: 28347450

[ref48] Houston T. K. Allison J. J. (2002). Users of internet health information: differences by health status. J. Med. Internet Res. 4:E7. doi: 10.2196/jmir.4.2.e7, PMID: 12554554 PMC1761934

[ref49] Janz N. K. Becker M. H. (1984). The health belief model: A decade later. Health Educ. Q. 11, 1–47. doi: 10.1177/109019818401100101, PMID: 6392204

[ref50] Jarvenpaa S. L. Tractinsky N. Saarinen L. (1999). Consumer Trust in an Internet Store: A cross-cultural validation. J. Comput.-Mediat. Commun. 5:JCMC526. doi: 10.1111/j.1083-6101.1999.tb00337.x

[ref51] Johnston A. C. Warkentin M. (2010). Fear appeals and information security behaviors: an empirical study. MIS Q. 34, 549–566. doi: 10.2307/25750691

[ref52] Khan G. F. Sarstedt M. Shiau W.-L. Hair J. F. Ringle C. M. Fritze M. P. (2019). Methodological research on partial least squares structural equation modeling (Pls-Sem). Internet Res. 29, 407–429. doi: 10.1108/IntR-12-2017-0509

[ref53] Kim K. K. Prabhakar B. (2004). Initial trust and the adoption of B2c E-commerce: The case of internet banking. ACM sigmis database 35, 50–64. doi: 10.1145/1007965.1007970

[ref54] Klein K. J. Sorra J. S. (1996). The challenge of innovation implementation. Acad. Manag. Rev. 21, 1055–1080. doi: 10.5465/amr.1996.9704071863

[ref55] Knitza J. Simon D. Lambrecht A. Raab C. Tascilar K. Hagen M.. (2020). Mobile Health Usage, Preferences, Barriers, and Ehealth Literacy in Rheumatology: Patient Survey Study. JMIR mHealth and uHealth 8:e19661. doi: 10.2196/19661, PMID: 32678796 PMC7450373

[ref56] Lagoe C. Atkin D. (2015). Health anxiety in the digital age: an exploration of psychological determinants of online health information seeking. Comput. Hum. Behav. 52, 484–491. doi: 10.1016/j.chb.2015.06.003

[ref57] Lee J.-H. (2016). Future of the smartphone for patients and healthcare providers. Health. Inform. Res. 22, 1–2. doi: 10.4258/hir.2016.22.1.1, PMID: 26893944 PMC4756052

[ref58] Li X. Hsieh J. P.-A. Rai A. (2013). Motivational differences across post-acceptance information system usage behaviors: an investigation in the business intelligence systems context. Inf. Syst. Res. 24, 659–682. doi: 10.1287/isre.1120.0456

[ref59] Lim E. T. Tan C.-W. Cyr D. Pan S. L. Xiao B. (2012). Advancing public trust relationships in electronic government: The Singapore E-filing journey. Inf. Syst. Res. 23, 1110–1130. doi: 10.1287/isre.1110.0386

[ref60] Lu J. (2014). Are personal innovativeness and social influence critical to continue with Mobile commerce? Internet Res. 24, 134–159. doi: 10.1108/IntR-05-2012-0100

[ref61] Lu J. Liu C. Yu C.-S. Wang K. (2008). Determinants of accepting wireless Mobile data Services in China. Inf. Manag. 45, 52–64. doi: 10.1016/j.im.2007.11.002

[ref62] McFarlin D. B. Sweeney P. D. (1992). Distributive and procedural justice as predictors of satisfaction with personal and organizational outcomes. Acad. Manag. J. 35, 626–637.

[ref63] McKinley C. J. (2009). Investigating the influence of threat appraisals and social support on healthy eating behavior and drive for thinness. Health Commun. 24, 735–745. doi: 10.1080/10410230903264303, PMID: 20183382

[ref64] McKinley C. J. Ruppel E. K. (2014). Exploring how perceived threat and self-efficacy contribute to college students’ use and perceptions of online mental health resources. Comput. Hum. Behav. 34, 101–109. doi: 10.1016/j.chb.2014.01.038

[ref65] Meng F. Guo X. Peng Z. Lai K.-H. Zhao X. (2019a). Investigating the adoption of Mobile health services by elderly users: trust transfer model and survey study. JMIR Mhealth Uhealth 7:e12269. doi: 10.2196/12269 30622092 PMC6329414

[ref66] Meng F. Guo X. Peng Z. Ye Q. Lai K.-H. (2021). Trust and Elderly Users’ Continuance Intention Regarding Mobile Health Services: The Contingent Role of Health and Technology Anxieties. Inform. Technol. People 35, 259–280. doi: 10.1108/ITP-11-2019-0602

[ref67] Meng F. Guo X. Peng Z. Zhang X. Vogel D. (2019b). The routine use of Mobile health Services in the Presence of health consciousness. Electron. Commer. Res. Appl. 35:100847. doi: 10.1016/j.elerap.2019.100847

[ref68] Meng F. Guo X. Peng Z. Zhang X. Vogel D. (2020). A 2020 perspective on “the routine use of Mobile health Services in the Presence of health consciousness”. Electron. Commer. Res. Appl. 40:100931. doi: 10.1016/j.elerap.2020.100931

[ref69] Morris M. G. Venkatesh V. (2000). Age differences in technology adoption decisions: implications for a changing work force. Pers. Psychol. 53, 375–403. doi: 10.1111/j.1744-6570.2000.tb00206.x

[ref70] Nunes A. Limpo T. Castro S. L. (2019). Acceptance of Mobile Health Applications: Examining Key Determinants and Moderators. Front. Psychol. 10:2791. doi: 10.3389/fpsyg.2019.02791 31920836 PMC6914844

[ref71] Okazaki S. Blas S. S. Castañeda J. A. (2015). Physician’s adoption of Mobile health monitoring Systems in Spain: competing models and impact of prior experience. J. Electron. Commer. Res. 16:194. doi: 10.4018/978-1-7998-8052-3.ch026

[ref72] Oliveira C. Pereira A. Vagos P. Nóbrega C. Gonçalves J. Afonso B. (2021). Effectiveness of Mobile App-Based Psychological Interventions for College Students: A Systematic Review of the Literature. Front. Psychol. 12:647606. doi: 10.3389/fpsyg.2021.647606 34045994 PMC8144454

[ref73] Or C. K. Karsh B.-T. (2009). A systematic review of patient acceptance of consumer health information technology. J. Am. Med. Inform. Assoc. 16, 550–560. doi: 10.1197/jamia.M2888, PMID: 19390112 PMC2705259

[ref74] Palvia P. (2009). The role of trust in E-commerce relational exchange: a unified model. Inf. Manag. 46, 213–220. doi: 10.1016/j.im.2009.02.003

[ref75] Pavlou P. A. (2003). Consumer acceptance of electronic commerce: integrating trust and risk with the technology acceptance model. Int. J. Electron. Commer. 7, 101–134. doi: 10.1080/10864415.2003.11044275

[ref76] Podsakoff P. M. MacKenzie S. B. Lee J.-Y. Podsakoff N. P. (2003). Common method biases in behavioral research: a critical review of the literature and recommended remedies. J. Appl. Psychol. 88:879. doi: 10.1037/0021-9010.88.5.879 14516251

[ref77] Prentice-Dunn S. Rogers R. W. (1986). Protection motivation theory and preventive health: Beyond the health belief model. Health Educ. Res. 1, 153–161. doi: 10.1093/her/1.3.153

[ref78] Rai A. Chen L. Pye J. Baird A. (2013). Understanding determinants of consumer Mobile health usage intentions, assimilation, and channel preferences. J. Med. Internet Res. 15:e149. doi: 10.2196/jmir.2635 23912839 PMC3742412

[ref79] Reychav I. Beeri R. Balapour A. Raban D. R. Sabherwal R. Azuri J. (2019). How reliable are self-assessments using Mobile Technology in Healthcare? The effects of technology identity and self-efficacy. Comput. Hum. Behav. 91, 52–61. doi: 10.1016/j.chb.2018.09.024

[ref80] Rogers R. W. (1975). A protection motivation theory of fear appeals and attitude Change1. Aust. J. Psychol. 91, 93–114. doi: 10.1080/00223980.1975.9915803, PMID: 28136248

[ref81] Rosenstock I. M. (1974). Historical origins of the health belief model. Health Educ. Monogr. 2, 328–335. doi: 10.1177/109019817400200403 299611

[ref82] Shareef M. A. Kumar V. Kumar U. (2014). Predicting Mobile health adoption behaviour: A demand side perspective. J. Cust. Behav. 13, 187–205. doi: 10.1362/147539214X14103453768697

[ref83] Shiau W.-L. Chau P. Y. (2016). Understanding behavioral intention to use a cloud computing classroom: a multiple model comparison approach. Inf. Manag. 53, 355–365. doi: 10.1016/j.im.2015.10.004

[ref84] Shiau W.-L. Sarstedt M. Hair J. F. (2019). Internet research using partial least squares structural equation modeling (Pls-Sem). Internet Res. 29, 398–406. doi: 10.1108/IntR-10-2018-0447

[ref85] Sun Y. Wang N. Guo X. Peng Z. (2013). Understanding the acceptance of Mobile health services: a comparison and integration of alternative models. J. Electron. Commer. Res. 14, 183–200. doi: 10.1080/15332969.2017.1289791

[ref86] Sundaram S. Schwarz A. Jones E. Chin W. W. (2007). Technology use on the front line: how information technology enhances individual performance. J. Acad. Mark. Sci. 35, 101–112. doi: 10.1007/s11747-006-0010-4

[ref87] te Poel F. Baumgartner S. E. Hartmann T. Tanis M. (2016). The curious case of Cyberchondria: a longitudinal study on the reciprocal relationship between health anxiety and online health information seeking. J. Anxiety Disord. 43, 32–40. doi: 10.1016/j.janxdis.2016.07.009, PMID: 27497667

[ref88] Thatcher J. B. McKnight D. H. Baker E. W. Arsal R. E. Roberts N. H. (2011). The role of Trust in Postadoption it Exploration: An empirical examination of knowledge management systems. IEEE Trans. Eng. Manag. 58, 56–70. doi: 10.1109/TEM.2009.2028320

[ref89] Venkatesh V. Morris M. G. Davis G. B. Davis F. D. (2003). User acceptance of information technology: toward a unified view. MIS Q. 27, 425–478. doi: 10.2307/30036540

[ref90] Venkatesh V. Speier C. Morris M. G. (2002). User acceptance enablers in individual decision making About technology: toward an integrated model. Decis. Sci. 33, 297–316. doi: 10.1111/j.1540-5915.2002.tb01646.x

[ref91] Witte K. (1992). Putting the fear Back into fear appeals: The extended parallel process model. Commun. Monogr. 59, 329–349. doi: 10.1080/03637759209376276

[ref92] Wood C. S. Thomas M. R. Budd J. Mashamba-Thompson T. P. Herbst K. Pillay D.. (2019). Taking connected Mobile-health diagnostics of infectious diseases to the field. Nature 566, 467–474. doi: 10.1038/s41586-019-0956-2, PMID: 30814711 PMC6776470

[ref93] Wu L. Li J.-Y. Fu C.-Y. (2011). The adoption of Mobile healthcare by Hospital’s professionals: an integrative perspective. Decis. Support. Syst. 51, 587–596. doi: 10.1016/j.dss.2011.03.003

[ref94] Xiao N. Sharman R. Rao H. R. Upadhyaya S. (2014). Factors influencing online health information search: An empirical analysis of a National Cancer-Related Survey. Decis. Support. Syst. 57, 417–427. doi: 10.1016/j.dss.2012.10.047

[ref95] Xue L. Yen C. C. Chang L. Chan H. C. Tai B. C. Tan S. B.. (2012). An exploratory study of ageing Women’s perception on access to health informatics via a Mobile phone-based intervention. Int. J. Med. Inform. 81, 637–648. doi: 10.1016/j.ijmedinf.2012.04.008, PMID: 22658778

[ref96] Zarmpou T. Saprikis V. Markos A. Vlachopoulou M. (2012). Modeling users’ acceptance of Mobile services. Electron. Commer. Res. 12, 225–248. doi: 10.1007/s10660-012-9092-x

[ref97] Zhang X. Guo X. Ho S. Y. Lai K.-H. Vogel D. (2020). Effects of Emotional Attachment on Mobile Health-Monitoring Service Usage: an Affect Transfer Perspective. Inf. Manag. 58:103312. doi: 10.1016/j.im.2020.103312

[ref98] Zhao L. Lu Y. Gupta S. (2012). Disclosure intention of location-related information in location-based social network services. Int. J. Electron. Commer. 16, 53–90. doi: 10.2753/JEC1086-4415160403

[ref99] Zhao Y. Ni Q. Zhou R. (2017). What factors influence the Mobile health service adoption? a Meta-analysis and the moderating role of age. Int. J. Inf. Manag. 43, 342–350. doi: 10.1016/j.ijinfomgt.2017.08.006

